# State-of-the-art approaches in the investigation of human seminal bacteriome using metagenomic methods

**DOI:** 10.3389/frph.2025.1557912

**Published:** 2025-06-05

**Authors:** Jan Hofman, Petra Brenerova, Petra Borilova Linhartova

**Affiliations:** ^1^ RECETOX, Faculty of Science, Masaryk University, Brno, Czechia; ^2^ Department of Histology and Embryology, Faculty of Medicine, Masaryk University, Brno, Czechia

**Keywords:** methodology, bacteriome, ejaculate, sperm, semen, bacteriospermia, spermiogram, fertility

## Abstract

Although the understanding of the causes of infertility is the key to its successful treatment, recent studies have shown that as many as 50% of male-caused infertility cases are considered idiopathic. The microbial colonization of the male reproductive system was shown to be associated with reduced male reproductive fitness. Investigation of the seminal microbiome, however, remains challenging. This article aimed to improve this situation by creating the first comprehensive review of literature on the metagenomic methods (including the pre-analytical and analytical approaches) used in the research on human seminal bacteriome (total bacterial DNA in the matrix), published in 2018–2024. A total of 29 studies addressing the analysis of the human seminal bacteriome were identified. The analysis typically involved DNA extraction from the supernatant using commercial kits, amplification of the gene for 16S rRNA, and sequencing of amplicons. Where the separation of seminal plasma was performed, centrifugation was the dominant method used for this purpose. The significant heterogeneity in individual steps of methodological approaches in the analysis of the human seminal bacteriome complicates the comparison of results among studies and the establishment of standard procedures, hindering clinical advancements. For this reason, a protocol for the analysis of the human seminal plasma bacteriome is proposed here, which could lead to improved comparability of results among studies and make future research more efficient. This protocol is founded on rigorous quality control measures, compliance with the WHO laboratory manual for sample collection, extensive pretreatment involving mechanical and enzymatic lysis, DNA extraction using the QIAamp DNA Mini Kit (Qiagen), and short-read sequencing conducted on the MiSeq platform (Illumina).

## 1 Introduction

Since the early 1950s, a noticeable decline in fertility rates across Europe has been observed, irrespective of cultural or social contexts (1). A surge in research focused on reproductive health has followed, intending to shed light on this phenomenon. Recent meta-analyses have reinforced the hypothesis that the seminal microbiota plays a crucial role in reproductive health (2). As a part of this effort, the reproductive microbiome, particularly the bacteriome, i.e., the total bacterial genetic information in a matrix (in this case, seminal plasma) at a given time, has emerged as a possible culprit of the decline. Infections of the urogenital tract directly associated with its microbial colonization are responsible for approximately 15% of male infertility cases (3–6). However, this number should not be regarded as definitive, as research (in particular) on asymptomatic infections, remains limited, and such infections are currently regarded as having minimal clinical significance (7, 8). On the other hand, certain bacteria have been associated with various mechanisms that negatively impact reproductive capacity. These mechanisms include the direct adhesion of bacteria to spermatozoa (9–11), effects mediated by their products (12), or adverse outcomes resulting from the increased presence of leucocytes (13–16) despite the absence of clinical manifestations. An improved understanding of the interactions between the seminal fluid and spermatozoa could lead to better identification of infections and, in effect, the possibility of successful treatment. In general, symptoms tend to increase with increasing bacterial abundance (13, 17–19). Conversely, reduced diversity of seminal bacteriome is generally considered a sign of dysbiosis (20). The interest in this field has increased over the last decade, with the current experimental studies primarily focusing on the correlation between sperm quality and bacteriome diversity and composition (21–44).

With this increasing intensity of research on microbial colonization of the male reproductive tract, there is an urgent need to evaluate and standardize methodological approaches to enhance the accuracy of microbiome analyses. The advent of next-generation sequencing (NGS) pushed traditional microbiological examination methods using culture media and biochemical tests into the background for research purposes, although they still hold an important place in clinical practice. Nevertheless, Mardanov et al. reported that culture methods can detect only approximately 1% of all microbial taxa (45). This, in conjunction with the problematic culture of anaerobic bacteria (46) that are ever-present in seminal bacteriome (17, 22, 34), makes these methods unsuitable for the analysis of bacteriome in a matrix as complex as seminal fluid. Therefore, the authors of this review decided to focus on culture-independent methods used for bacteriome research. Presently, 16S rRNA amplicon sequencing is the predominant method employed in the study of the bacteriome due to a combination of analytical precision and cost-effectiveness. Such analysis of the human seminal bacteriome can be supplemented or substituted with targeted analyses of bacterial DNA, paving the way for clinical applications in diagnosing reproductive tract diseases and predicting male fertility.

This review article aims to (i) summarize the pre-analytical and analytical approaches used in the metagenomic analyses of the human seminal bacteriome, (ii) critically evaluate their pros and cons, and (iii) propose a workflow for the metagenomic analyses of seminal bacteriome reflecting the current state of the art, which could be used for future experimental studies. All of this should contribute to more accurate research on the seminal bacteriome in male reproductive health (while, of course, leaving space for individual researchers to adjust the workflow to their needs). This would increase the legitimacy of the studies performed in this field (47) as well as the comparability of results for future meta-analyses.

## 2 Literature research

**Figure 1:**
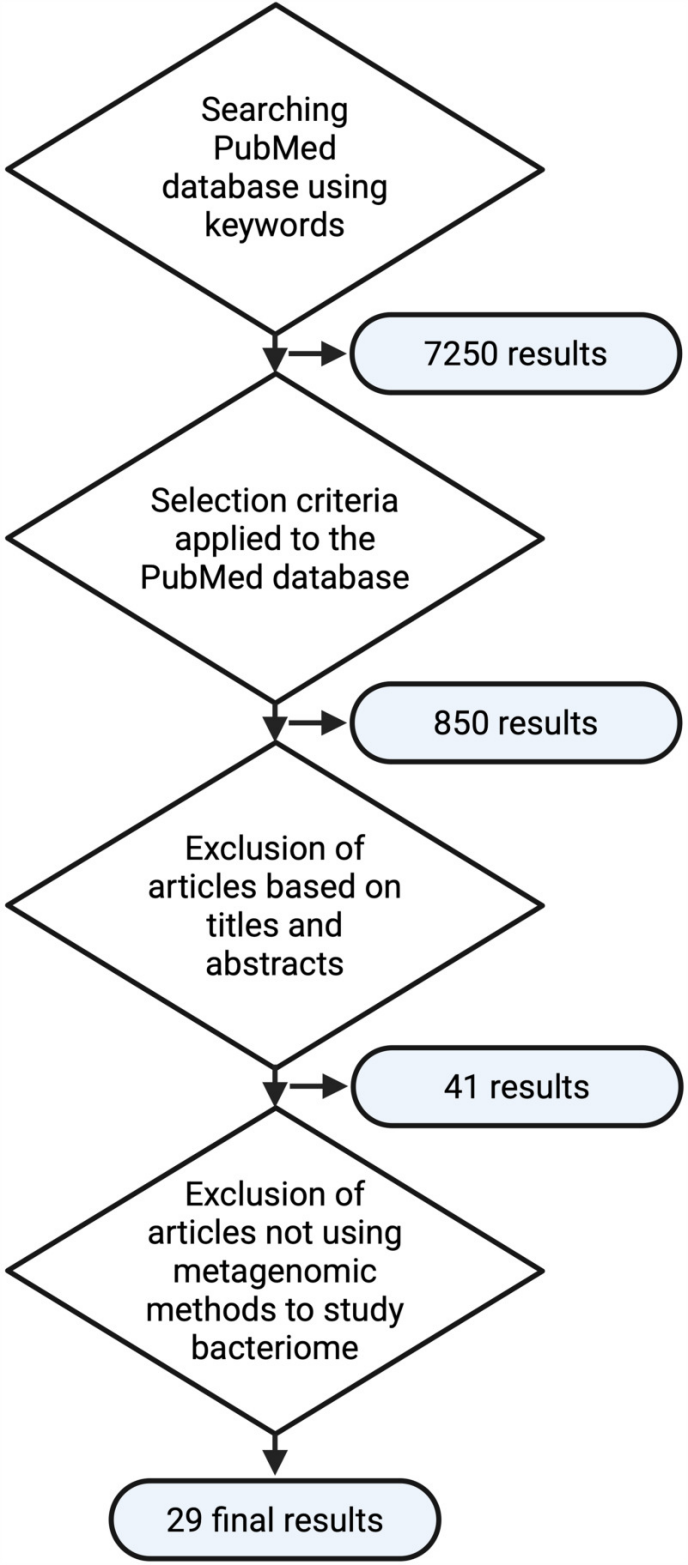
Flowchart of the methodological approach used in the literature review (created with bioRender.com).

The PubMed database was searched using keywords “(seminal OR sperm OR ejaculate) AND (microbiome OR microbiotic OR microbiotix OR microbiota OR microbiomes OR bacterium OR microbiology OR bacterial) OR (bacteriospermia)”, with additional criteria for article selection as follows: (i) only original articles, (ii) published from 2018 onwards to ensure the up-to-date metagenomic methods and knowledge in the dynamic field of molecular biology, and (iii) conducted on human subjects. Subsequently, titles and abstracts were screened to identify relevant articles and only relevant studies that describe the metagenomic analysis of the complex bacteriome (not just the analysis of individual clinically significant species) were used as information sources. This methodological algorithm is illustrated in Figure 1.

## 3 Pre-analytical and analytical approaches used in the metagenomic analyses of the human seminal bacteriome

The initial literature search yielded 850 results. After scanning the titles and abstracts, 41 relevant original articles (13, 14, 19, 21–44, 46, 48–60) listed in the PubMed database and published since 2018 were identified. Twelve of these studies (13, 14, 19, 48–50, 52–57), however, did not analyze the microbiome but used microbiological and analytical methods to determine cultured bacteria; these studies with an alternative approach using inoculation of the sample on bacteriological media and analysis of the resulting colonies [mass spectrometry MALDI-TOF (48–50), VITEK II (13, 14, 19, 52, 54), or microscopic analysis (13, 14, 19, 48, 50, 53–57)] were excluded from further analysis, yielding a final set of 29 studies. The predominant focus of these studies was to explore the relationship between the seminal bacteriome and various health conditions, particularly infertility (21–25, 27–31, 33–39, 41–44, 46, 58, 59), prostatitis (40, 42), vasectomy (51), or cancerous diseases (26, 32). One study focused on the forensic evaluation of ejaculate (60). The sample sizes in these studies varied greatly, ranging from five to 285 volunteers/patients. The median size of the study sample was 56 participants.

### 3.1 Pre-analytical phase—collection of ejaculate sample, its processing, and storage

Out of the final set of 29 studies, the samples were collected through ejaculation into a sterile container in 27 studies. Depending on the design, this was in some studies supplemented by testicular sperm extraction (TESE) (21, 26, 27), percutaneous epididymal sperm aspiration (PESA) (21), or micro epididymal sperm aspiration (MESA) (42). These modified collection methods were used due to obstructive azoospermia or the need to collect a sample from a specific part of the male reproductive tract.

Sample collection through ejaculation into a sterile container was performed, with few exceptions, according to the principles recommended by the World Health Organization (WHO) (61), which entails (a) minimizing temperature fluctuation and time before and during analysis; (b) 2–7 days of sexual abstinence before the collection (maintaining a constant interindividual duration); (c) urination, washing the glans penis and hands with soap, rinsing everything well and drying before the actual collection; (d) collecting the entire ejaculate fraction into a sterile plastic or glass container tempered to 20–37°C; (e) processing the samples within three hours at room temperature; (f) placing the samples into an environment with a temperature of 37°C as soon as possible for precise evaluation of the liquefaction time; and (g) exclusive use of sterile laboratory material, and classification of all samples as biohazard.

Immediately after collection, the liquefaction of the ejaculate (i.e., the rate of proteolytic processes transforming a gel-like ejaculate into a watery one) at 37°C was usually evaluated. The evaluation of liquefaction must be completed within one hour of collection (61), and processing of the ejaculate sample itself immediately follows (within three hours at room temperature). Alternatively, the ejaculate samples were stored using cryopreservation methods—usually at −80°C (21, 27, 28, 38, 44), alternatively at −20°C (33, 46); in one study, samples were frozen at −20°C without any cryopreservative and then transferred to storage at −196°C (34).

Processing of the ejaculate includes separation of seminal plasma by centrifugation (22, 32, 38, 46), which partially removes spermatozoa, and, thus, human genetic information, from the sample. This step is vital in *in vitro* fertilization procedures; from this perspective, including this step is highly suitable if aiming to examine the microbiome directly associated with these procedures. The process takes place at room temperature. Unfortunately, centrifugation parameters were mentioned in three studies only and the protocol for separating spermatozoa from seminal plasma was not specified in any of them. Where centrifugation parameters were provided, the relative centrifugal forces were 800 g for 15 minutes with repeated centrifugation of the supernatant at 10,000 g for 10 minutes (32), and 7,000 g for 10 minutes (22). Gradient centrifugation was used in two studies (38, 46). Samples of seminal plasma for subsequent extraction of microbial DNA were stored at −80°C (22, 32) or were immediately used in the analytical phase.

Centrifugation was in two instances supplemented with the addition of dithiothreitol (DTT; 43 mM) as a reducing agent (23, 33). The purpose of DTT is to disrupt disulfide bridges in the protein structure, thus lysing the nuclear membrane of incompletely removed spermatozoa. This leads to the release of human DNA into the sample; it is, however, necessary to emphasize that unless whole-genome sequencing is used as the subsequent analysis, this poses no problem for bacterial identification using other methods (such as 16S rRNA PCR).

### 3.2 Analytical phase—extraction of microbial DNA from human seminal plasma

In all included bacteriome studies, the analysis was based on evaluating bacterial DNA (21–44, 46, 51, 58–60). In 22 cases, a commercial kit based on the spin column principle (21–28, 30, 31, 34, 35, 37–42, 46, 58–60) was used. Alternative solutions included the phenol-chloroform extraction (36, 43, 44), the Trizol LS reagent protocol (Invitrogen, USA) (32), a protocol utilizing cetrimonium bromide (CTAB) (29), and the NucliSENS easyMAG system (BioMèrieux, USA) (33) working on the principle of binding magnetic silica particles to the nucleic acid and their subsequent separation in the magnetic field.

The extraction procedures for obtaining microbial DNA from seminal plasma can be modified or combined. To achieve better cell lysis prior to the DNA extraction, pre-treatment using proteinase K with phosphate buffer and/or bead beating methods using glass, ceramic, or metal particles was utilized in eleven studies (21, 23, 25, 27–30, 34, 39, 41, 59).

The QIAamp DNA mini kit (Qiagen, USA/Germany/Switzerland) was the most commonly used one (22, 23, 35, 46). The manufacturer's instructions were typically adhered to, except for one study where (due to the low total amount of bacterial DNA in the sample), the amounts of reagents were changed while maintaining ratios (27). In another study, an additional step of washing during centrifugation (8,000 g; 1 min) prior to the use of the ZymoBIOMICS DNA Microprep kit (Zymo Research, USA) was added (59). The CTAB method was used in one study (29), the Trizol LS Reagent protocol (Invitrogen, USA) for the extraction of small RNA in another (32).

In several studies, Tris buffer and proteinase K were used for sample pre-treatment before phenol-chloroform extraction (36, 43, 44). In one study (32), DNA extraction was preceded by sample homogenization and cell lysis using the TissueLyser LT (Qiagen) homogenizer with the addition of 400 μl of Tris buffer.

Besides the aforementioned methods, in one case, the NucliSENS® easyMAG® (Biomerieux, USA) system was used according to the manufacturer's instructions (33).

### 3.3 Analytical phase—analysis of DNA extracted from human seminal plasma

The methods of bacteriome analysis used in the analyzed studies differed in their ability to determine taxonomic units. In four studies, PCR served for qualitative and/or quantitative determination of bacterial DNA. These included single-target qPCR (24, 25, 46) and multiplex qPCR (25, 31). Multiplex qPCR was used in studies targeting the analysis of specific difficult-to-culture bacteria, especially from the Mycoplasmataceae family, as well as *Gardenella vaginalis, Sneathia sanguinegens, Bacteroides fragilis*, and *Megasphaera* (25, 31). Amplification of the gene for 16S rRNA followed by NGS was the most common method (23 studies) (22, 23, 27–30, 32–41, 43, 44, 46, 51, 58–60). In some cases, it was supplemented with real-time (RT) qPCR (30, 33). In one study, these PCR products were analyzed using denaturing high-performance liquid chromatography (DHPLC) (42).

When amplifying the gene for 16S rRNA for subsequent sequencing (22, 23, 27–30, 32–41, 43, 44, 46, 51, 58–60), conditions were set depending on the chosen primers and master mix requirements. The choice of variable regions for PCR amplification itself presents a source of a certain variability among studies. Predominantly, PCRs focused on the V3–V4 (27, 28, 35, 37, 38, 40, 60) and V1–V9 regions (34, 59), although some researchers focused on the V3 (33), V4 (21, 30), V6 (58), V3–V6 (22), V3-V5 (26), V1-V3 (41), V1–V2 (23, 29, 39, 51), V2–V3 (46), or V4–V6 (44) regions. Nested PCR was used in two studies to reduce non-specific primer binding to DNA sequences outside the observed segments (26, 33). In both instances, the DNA was amplified to be sequenced [pyrosequencing and sequencing on the Ion Torrent PGM platform (Life Technologies, USA)]. One of these studies employed the V3–5 region for amplification (26), the other used just the V3 area (33).

Before the actual analysis, an additional step of amplicon purification was mentioned in 15 studies. AMPure XP beads (Beckman Coulter, Italy/USA) in one (23, 27, 32, 37–39, 44, 60) or two (26) cycles were the most commonly used of these methods. Other techniques included AxyPrep DNA Gel extraction kit (Axygen) (35), QIAquick column (Qiagen, USA) (51), NucleoMag NGS Clean-up (Macherey-Nagel, France) (41), and, in two cases, Zymo-Spin IC column (Zymo Research, USA) (34, 59).

Sequencing techniques play a dominant role in seminal microbiome analysis. Illumina products are the most commonly used for this purpose, including platforms such as the MiSeq system (fourteen studies) (23, 27, 28, 30, 34–36, 38–41, 44, 51, 60), HiSeq 2000 (two studies) (21, 58), HiSeq 2500 (one study) (29), HiSeq 4000 (one study) (32), and NovaSeq 6000 (Illumina Co., USA) (one study) (37). Besides Illumina products, sequencing using MinION (Oxford Nanopore Technologies) was employed as an alternative in one case (59). Pyrosequencing was applied in two studies, using the platforms Roche 454-GS Junior (Roche, USA) (26) and Roche 454 FLX (Roche, USA) (46). The Ion Torrent PGM (Thermo Fisher Scientific, USA) procedure was employed in two studies (22, 33).

Various library preparation kits were used to prepare libraries, including the NGSgo kit (GenDx, Netherlands) (34), CATS small RNA Quick-16S NGS (Diagenode, Belgium) (32), Quick-16S NGS library prep kit (Zymo Research, USA) (36), Ion Plus Fragment Ion PGM Hi-Q (Thermo Fisher Scientific, USA) (33), or Nextera XT (Illumina, USA) (27, 28, 41). Five studies used, in addition to the above, a control sequencing library, specifically PhiX (Illumina) (23, 32, 38, 41, 44). The final analysis of outputs also varied according to the respective bioinformatics pipelines.

**Figure 2:**
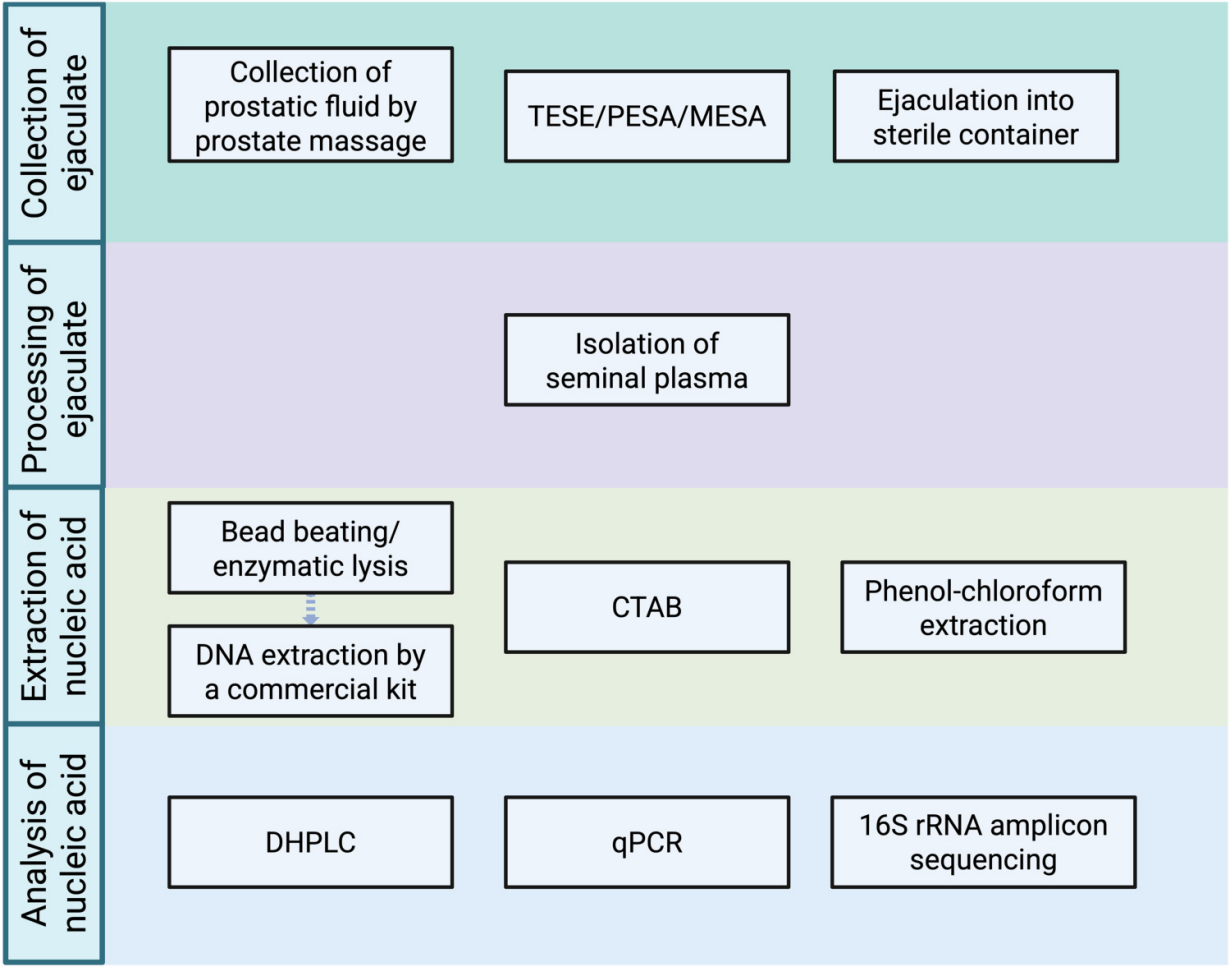
Methods previously used in individual steps of the analysis of human seminal bacteriome (created with bioRender.com). (TESE, testicular sperm extraction; PESA, percutaneous epididymal sperm aspiration; MESA, microsurgical epididymal sperm aspiration; CTAB, cetrimonium bromide method; qPCR, quantitative polymerase chain reaction; DHPLC, denaturing high-performance liquid chromatography.).

Based on the literature review, the general workflow for analyses of seminal bacteriome is shown in Figure 2, and methods used in each study are highlighted in Supplementary Table 1 within Supplementary 1.

## 4 Critical evaluation of the approaches used in the metagenomic analyses of the human seminal bacteriome

### 4.1 The WHO manual on semen collection and its application in studying the seminal microbiome

The WHO laboratory manual for the examination and processing of human semen has become the gold standard in the field (61). However, it is necessary to keep in mind that the manual primarily focuses on sperm quality assessment and processing protocols, not on the analysis of seminal plasma. In the reviewed studies, sample collection was consistently executed through ejaculation into a sterile container, adhering to WHO guidelines within the environments of reproductive health clinics.

WHO guidelines recommend the start of the microbial analysis or freezing of the samples within 3 hours of collection (61). This seems reasonable as the collection of samples usually happens in external infrastructures such as *in vitro* fertilization (IVF) clinics and adhering to WHO recommendations generally seems to be a good way of ensuring standardization across studies. This, of course, does not preclude researchers from more rapid processing, which could be an even better option if feasible. However, studies examining bacteriome change in skin swabs and stool samples have not found any statistically significant change even after 24 hours (62, 63). The only study focusing on the temporal change in seminal samples focused on forensic analysis, considering a much longer time frame (weeks) (64). Still, as a precaution against the introduction of error, it is reasonable to keep the time from sampling to analysis/storage constant among samples within a single study. Similarly, collecting samples at the same time of day could also be recommended to account for any possible intrapersonal diurnal changes in the microbiome composition (although no studies have examined this in seminal samples so far). Lastly, repeated thawing and re-freezing of samples is not recommended (65).

The length of sexual abstinence before collection presents another aspect of data collection. WHO recommends 2–7 days of sexual abstinence prior to sampling (61). Shorter abstinence has been associated with decreasing bacterial abundance and diversity (48). Moreover, other factors play a role in changing the bacteriome composition with abstinence (48). Because of this, it is reasonable to keep the abstinence period preceding the sample collection as consistent as possible to ensure the same time for bacterial growth. This being said, the period should fall within the WHO-recommended time frame to support comparison among studies and to reflect the general clinical practice at IVF clinics. If choosing between shorter and longer abstinence within this timeframe, the longer time might be beneficial by leading to higher bacterial abundance, which could be advantageous considering that the ejaculate is a low-abundant matrix.

In the pre-analytical phase, the separation of the seminal plasma and microbial DNA from spermatozoa is an optional approach for minimizing the load of human DNA. However, the WHO guidelines lack a detailed methodological workflow for processing seminal plasma samples for microbiome analysis; to make things worse, most studies fail to specify their processing techniques. Various methods, such as magnet-activated cell sorting (MACS), discontinuous density gradient, or direct swim-up, are available for separating motile and morphologically normal spermatozoa (61). Nonetheless, these methods can significantly reduce sperm yield (66), rendering them unsuitable for simultaneous spermatozoa and microbiome analyses. Furthermore, any such manipulation of the sample may alter the bacterial composition. Reports of spermiogram analyses conducted according to WHO principles are not common in the included studies, despite the adherence to the general guideline. The analytical methods employed for the microbiome studies varied considerably compared to the more standardized sample collection procedures.

Samples obtained via TESE, PESA, and MESA methods possess unique characteristics that must be acknowledged during processing. For instance, the prostatic fluid´s role in ejaculate liquefaction is significant (67). If any ejaculate fraction is absent, the workflow needs to be adjusted to account for the physiological properties of the matrix. TESE, PESA, and MESA sampling methods are primarily suitable for microbiome analysis from specific regions of the male reproductive tract or in cases of obstructive azoospermia precluding traditional collection methods (42).

### 4.2 How to process an ejaculate sample and obtain seminal plasma for microbiome analysis?

Immediately after collection, the ejaculate sample must be liquefied to allow the extraction of the microbial genetic material. Liquefaction is a process facilitated by prostatic serine proteases capable of breaking down the (predominantly) fibrin matrix present in the ejaculate. The duration of liquefaction is a critical diagnostic criterion linked to spermiogram quality, with certain microbial taxa potentially influencing the speed and quality of this process (68). Maintaining the optimal conditions for liquefaction, particularly the temperature of 37°C immediately after collection, is essential. The liquefaction typically occurs within 15–30 minutes; failure of its completion within 60 minutes may indicate a pathological condition (61). Orbital movement (e.g., using an orbital mixer) can make the process more efficient.

To ensure representative sampling, the entire volume of the sample should be homogenized before further processing. This can be achieved through 15–30 s of swirling movement (manually or with an orbital mixer). Working with ejaculate can pose ethical complexities compared to working with seminal plasma alone, necessitating the removal of human DNA prior to nucleic acid extraction, although lysed spermatozoa do not interfere with the PCR amplification of the gene for 16S rRNA (21, 23–30, 33–44, 46, 51, 58–60). Centrifugation methods are commonly employed for this purpose, but there is considerable variability in parameters across studies, particularly regarding the relative centrifugal force (RCF). Temperature conditions are usually not described, with room temperature being the standard when mentioned.

Two primary centrifugation approaches are recognized: gradient centrifugation and simple centrifugation. However, only two reviewed studies reported the use of a density gradient (38, 46). Gradient centrifugation is more complex and costly; however, if using parameters according to the specific parameters outlined in the WHO manual (200–400 g for 15–20 minutes using 40% and 80% gradients) (61), a lower fraction of spermatozoa is separated from seminal plasma (note the different purpose of the WHO guideline). This, on the one hand, better prevents undesirable separation of the microbiota but, on the other, does not reliably remove spermatozoa and human DNA from the sample. However, as mentioned above, this does not necessarily pose a problem as from the perspective of 16S rRNA amplification, no separation of spermatozoa from the seminal plasma is necessary. Hence, if aiming to analyze the ejaculate itself, none of the centrifugation methods described in the literature can be recommended as the risk-to-benefit ratio is unreasonable. The risk of removing an unknown amount of bacteria with unknown composition is too high; moreover, the elimination of sperm cells is ineffective, leaving a significant amount of sperm cells as well as of free human DNA in the supernatant (69), which prevents meaningful metagenomic analysis anyway.

If the methodology (such as the utilization of a metagenomic approach) or external factors (such as ethical concerns) demand the removal of spermatozoa, it would be probably better to resort to methods that have been successfully used in other matrices, despite the fact that, with one exception, they have not yet been tested on ejaculate samples (which, in any case, calls for performing such methodological studies). Even there, however, it is necessary to be careful regarding the choice of the separation method. Filtration utilized, for example, for the depletion of epithelial cells from saliva (70) might be suboptimal as it cannot remove free DNA (69). Selective depletion of host DNA in seminal samples might be a more suitable approach. However, this approach has been sparsely tested on ejaculate samples. This depletion can be achieved by selective lysis of eukaryotic cell wall using reagents such as saponin, Triton X-100, or Tween 20 (71–73), followed by the degradation of free-floating DNA using either Dnase I (74, 75) or benzonase nuclease that has greater toleration to working conditions (69). The intricacy of this approach, however, lies in the composition of the sperm cell wall. Spermatozoa have a specific cell wall structure containing a higher percentage of polyunsaturated fatty acids (76), which makes the success of the lysis likely; so far, nevertheless, it has been verified in just a single study using the QIAmp DNA Microbiome kit (Qiagen, USA) (28). Moreover, it reportedly decreases bacterial abundance in low-abundant samples (69) and can be only applied on fresh samples (not frozen as freezing could cause the disruption of the bacterial cell wall as well).

Enzymatic depletion of eukaryotic DNA based on specific enzymes targeting methylated CpG regions after the DNA release from a cell is another (at least theoretically) possible option (77). The pitfall of this method, however, might lie in the specific way human DNA is stored within the spermatozoa. This DNA not only possesses characteristic methylome (78) but is also stored in a protamin-bound structure, which could complicate the process. However, no commercially available kit has been so far tested for this application, which, again, calls for methodological research in this area. Finally, the utilization of Oxford Nanopore MinION and Crisp-Cas9 technology represent promising concepts for the future; they need to be validated before use (69).

### 4.3 DNA extraction from human seminal plasma

The extraction of microbial nucleic acid from the sample is an essential step in the analysis of the human seminal microbiome. PCR-based amplification techniques surpass the culture-based techniques in allowing the detection of non-culturable microorganisms, thus providing a more comprehensive view of the microbial landscape within the seminal fluid.

The efficacy of microbial DNA extraction is influenced by several factors, including sample quality, microorganism lysis (often supplemented with bead beating), and the purification of genetic material from proteins and other substances.

The lysis of bacterial cells was suggested to be a more important step for maximizing the DNA yield than recovery steps (79–82). Utilizing enzymatic (83, 84) and/or mechanical lysis (81, 85, 86) was reported to yield higher amounts of bacterial DNA from the sample compared to no pretreatment. The combination of the aforementioned procedures is reported to slightly reduce the abundance of bacterial DNA; on the other hand, it improves the representativeness (and, therefore, the accuracy of subsequent determination) of microbiota in oral (83, 87) and gut (84) samples. The increase in bacterial DNA abundance after the use of enzymatic lysis is likely because of the ability of lysozymes to disrupt peptidoglycans of Gram-positive bacteria; on the other hand, the slight decrease in DNA abundance after the mechanical lysis is probably associated with a loss of a part of the sample during the procedure (83). A combination of several lysis enzymes (lysozyme, mutanolysin, staphilysin) seems to perform even better than the use of lysozyme alone (84). The use of the correct size of beads for mechanical lysis is also an important factor, as a size too big can reduce the amount of extracted DNA (84). It needs to be, however, mentioned that no methodological study directly comparing the use of multiple pretreatment techniques in ejaculate samples has been published so far.

The extraction of microbial DNA itself is typically performed using commercial kits, in some studies supplemented with a reducing agent (e.g., DTT) (23, 33) for better protein denaturation and removal. The selection of commercial DNA extraction kits is often guided by factors such as ease of use, reproducibility, and compatibility with downstream applications. Due to these advantages, commercial kits have become a preferred method for investigating the seminal bacteriome. However, the suitability of a specific kit depends on several factors, particularly the intended method of the extracted DNA evaluation. As discussed previously, kits that facilitate both the depletion of host (human) DNA and the extraction of high-molecular-weight DNA are advantageous for metagenomic applications. Gant et al. evaluated six commercial kits for their performance in host DNA depletion and suitability for long-read sequencing using the Oxford Nanopore MinION platform. The kits included the NucleoSpin Food Kit, Quick-DNA HMW MagBead Kit, ZymoBIOMICS DNA Miniprep Kit, QIAamp PowerFecal Pro Kit, Moss protocol, and DNAexpress Kit. Among these, the Quick-DNA HMW MagBead Kit was identified as the most suitable for this application (88). Notably, according to the manufacturer, this kit is compatible with a variety of biological fluids, further supporting its versatility for microbiome research. In another comparative study, Wright et al. evaluated the performance of several commercial DNA extraction kits—Qiagen DNeasy PowerSoil Pro Kit, HostZERO Microbial DNA Kit, PureLink Microbiome Kit, and Qiagen DNeasy Blood and Tissue Kit—using vaginal samples as the test matrix (89). Their findings revealed that all tested kits introduced some level of bias in microbial community profiles. However, the HostZERO Microbial DNA Kit was identified as the least suitable for vaginal samples due to its pronounced impact on microbial composition.

No studies comparing the performance of multiple kits for extracting DNA for microbiome profiling using short-read 16S rRNA amplicon sequencing in ejaculate samples have been published so far. However, cautious extrapolation from studies on vaginal and cervical microbiome may provide some information as sexual partners were shown to share approximately 56% of their genital microbiota (30), including many clinically relevant pathogens. In vaginal microbiome studies, the DNeasy Blood and Tissue Kit has been reported to yield a higher DNA quantity compared to the MoBio PowerSoil Kit, although the latter provided greater microbial diversity in the resulting data (90). This highlights a trade-off between DNA yield and community diversity that must be considered when selecting a kit for specific research objectives. Shibata et al. conducted a comparative analysis of several DNA isolation kits on cervical microbiota samples, including the ZymoBIOMICS DNA Miniprep Kit, QIAamp PowerFecal Pro DNA Kit, QIAamp DNA Mini Kit, and the IndiSpin Pathogen Kit (91). Their findings indicated that all tested kits produced comparable results in terms of microbiome composition, suggesting a degree of interchangeability for studies involving cervical samples. In the context of low-biomass samples from the urogenital tract, such as urine, multiple studies have identified the DNeasy Blood and Tissue Kit to be the most suitable option for DNA extraction. Its effectiveness in such matrices has been consistently demonstrated (92, 93), further supporting its potential application in studies of the seminal microbiome.

When considering DNA extraction kits for long-read sequencing of the seminal microbiome, products from Zymo Research appear to be particularly suitable. On a sample consisting of Gram-positive *Bacillus subtilis* and Gram-negative *Escherichia coli*, the Quick-DNA HMW MagBead Kit was reported to be the most effective among several compared in a recent benchmarking study (88). In ejaculate samples, the ZymoBIOMICS DNA Miniprep Kit has been recently successfully employed in the analysis of ejaculate samples (59).

To date, the QIAamp DNA Mini Kit has been the most commonly used extraction method for short-read 16S rRNA amplicon sequencing in seminal microbiome studies (22, 23, 35, 46). When comparing 16S rRNA amplicon sequencing in ejaculate and vaginal samples, it must be noted that ejaculate typically exhibits lower bacterial load but higher diversity than vaginal samples (30, 58, 94), which complicates a direct comparison of the results of different extraction methods. This makes it unclear whether higher-yielding kits, such as the DNeasy Blood and Tissue Kit, or a diversity-optimizing kit, such as the MoBio PowerSoil Kit, would be more appropriate for ejaculate samples. Moreover, the ejaculate microbiome shares significant bacterial overlap with the urinary microbiome (17), for which the DNeasy Blood and Tissue Kit has been repeatedly recommended, making this kit another promising candidate for seminal microbiome studies. At present, however, due to the limited number of comparative studies directly evaluating extraction methods in ejaculate samples, it is not yet possible to make definitive recommendations. However, based on current literature, the QIAamp DNA Mini Kit is the most commonly used kit and, therefore, can be considered a reasonable standard for ongoing research. Still, continued investigation is critically needed to determine the optimal extraction protocol for ejaculate samples, which would ultimately contribute to methodological standardization and improved reproducibility in the field.

### 4.4 Sequencing analyses of human seminal bacteriome

The conditions of PCR done on the microbial DNA preceding sequenation need to be chosen depending on the specific master mix and primers. The advent of long-read sequencing technologies has opened new possibilities for studying bacterial communities by allowing the amplification and analysis of the full-length gene for 16S rRNA, rather than focusing solely on its hypervariable regions. This approach enhances taxonomic resolution, particularly at the species level, while maintaining comparable accuracy at higher taxonomic levels such as genus and family (95–99). However, long-read sequencing remains technically demanding and requires the use of third-generation sequencing platforms such as PacBio (95) or Oxford Nanopore Technologies’ MinION (96, 100). To date, the number of studies investigating the use of sequencing platforms on seminal microbiome is severely limited. A comparison of the Illumina MiSeq and the ONT MinION platform reported no significant differences in the identification of abundant taxa between platforms (34, 59). However, differences in low-abundant taxa were observed, likely due to the disproportionate impact of sequencing errors on taxa with low relative abundance. Notably, both studies detected distinct bacterial community structures at the genus level, which may reflect sequencing biases intrinsic to each platform (101, 102). Still, looking forward, long-read sequencing appears to be a promising direction for seminal microbiome research.

Nevertheless, in short-read sequencing workflows, the choice of hypervariable region for PCR amplification remains an important consideration. Currently, no consensus exists on the optimal region, and different regions offer varying degrees of taxonomic resolution and bacterial coverage (103–107). Moreover, there are no comparative data specifically evaluating hypervariable regions in the context of ejaculate samples. When using short-read sequencing, it is well-established that no single hypervariable region can capture the full diversity of bacterial taxa (83). It was suggested that a correct combination of hypervariable regions could improve final results (108); however, multiple studies indicate that the impact of hypervariable region selection is generally less significant than that of DNA extraction methods (83, 109). Furthermore, the optimal region may vary depending on the biological sample (83, 104, 109–111).

As there are no studies directly addressing the performance of individual hypervariable regions in ejaculate samples, cautious extrapolation from vaginal microbiome research can help here as mentioned above. Even though the V1–V3 (112, 113) regions are the most commonly used in vaginal microbiome studies, the V3–V4 regions were shown to perform better than V1–V2 regions (112, 113).

Unlike in vaginal samples, the V3–V4 regions are currently the most frequently employed in ejaculate microbiome studies. Importantly, none of these studies reported issues regarding the resolution power of the V3–V4 region in practice. Given its consistent performance, widespread use, and supportive findings from related vaginal microbiome studies, it can be concluded that the V3–V4 region appears to be a suitable target for bacterial profiling of ejaculate samples. Nevertheless, long-read sequencing technologies offer superior taxonomic resolution and represent a promising advancement for future seminal microbiome research.

The analysis of microbial DNA is typically conducted following standardized recommendations by the manufacturers of the sequencing tools. The dominance of Illumina (used in more than 75% of studies performing sequencing analyses of seminal bacteriome) underscores the status of that company as the leader in (not only) seminal microbiome analysis.

Based on current evidence, the continued use of Illumina MiSeq for seminal microbiome studies can be recommended due to its established performance, broad adoption, and availability of validated protocols. Looking ahead, a gradual shift toward third-generation sequencing platforms can be anticipated, driven by improving error rates, enhanced protocols, and the superior resolution they offer. Nevertheless, it is true that platform selection is often determined not only by technical performance but also by availability, cost, and institutional bioinformatic support. Importantly, with the exception of two seminal microbiome studies, most comparative data cited here are derived from other body site microbiomes, highlighting the need for further research specifically targeting the seminal microbiome to optimize platform selection.

### 4.5 Quality control

Contamination remains a persistent concern in microbiome research, particularly in studies involving low-biomass samples, where the relative contribution of contaminants increases as the endogenous microbial signal decreases (114–116). Alarmingly, contaminants are frequently detected within laboratory reagents, especially DNA extraction kits, which can introduce exogenous bacterial DNA and compromise data integrity (115). Additional major sources of contamination include well-to-well cross-contamination during sample processing (117) and environmental contamination originating from laboratory surfaces and the surrounding air (115).

To mitigate contamination and improve laboratory reliability, several best practices have been established. These include regular environmental (surface wipe) testing, routine monitoring of sample positivity rates, incorporation of process controls, tracking of laboratory issues or complaints, and ensuring sufficient personnel training. The physical separation of workstations based on the workflow stage is another practice recommended to prevent cross-contamination (118). Additionally, randomization of sample placement on plates has been shown to reduce well-to-well contamination (117). Strict sterilization protocols and the use of laminar flow hoods or biosafety cabinets during sample handling should be considered essential components of contamination prevention, particularly in 16S rRNA amplicon sequencing workflows.

For each experiment, the use of negative and positive controls plays a crucial role in ensuring valid interpretation of results and prevention of erroneous conclusions (116, 119). Negative controls help identify environmental contamination, while positive controls or an internal standard ensure that a negative result is not due to procedural errors. These controls were widely utilized in the studies, both during microbial DNA extraction (23, 25–28, 34, 37–39, 59, 60) and during PCR amplification (25, 35, 38, 39, 41, 43, 44). In several papers, authors even resorted to using alternative analytical methods to validate their results and to rule out the inadequacy of their primary techniques (25, 59). The incorporation of MOCK microbial communities (MOCKs) as *in situ* positive standards is a strategy widely adopted for contamination control, assessment of protocol-associated biases, and validation of bioinformatic pipelines (116, 120, 121). MOCKs, composed of known microbial taxa, provide a benchmark for evaluating experimental and analytical steps, allowing researchers to detect unexpected taxa indicative of contamination and to gauge the reliability of sequencing and taxonomic assignment.

Post-sequencing computational tools also play a crucial role in identifying and removing contaminant sequences. Tools such as Decontam use statistical models to distinguish true biological signals from contaminants based on the frequency and prevalence across sample types (116, 122, 123), GRIMER (124) offers interactive tools for identifying potential sources of contamination and visualizing microbial profiles, while SourceTracker employs Bayesian methods to trace the origin of microbial communities, aiding in the attribution of contamination to environmental or reagent sources (116). The significance of these controls cannot be overstated, and the fact that some studies failed to report them does not necessarily imply non-use.

At this point, it is crucial to emphasize the importance of performing analyses that would provide a head-to-head comparison of the approaches currently employed in studying the human seminal bacteriome. Such a comprehensive evaluation would enhance analytical accuracy as well as the reliability and comparability of findings across studies. These efforts can pave the way for more robust conclusions and foster a deeper understanding of the human seminal microbiota, ultimately contributing to advancements in both basic and applied microbiological research.

## 5 Recommended workflow for the metagenomic analysis of human seminal bacteriome

**Figure 3:**
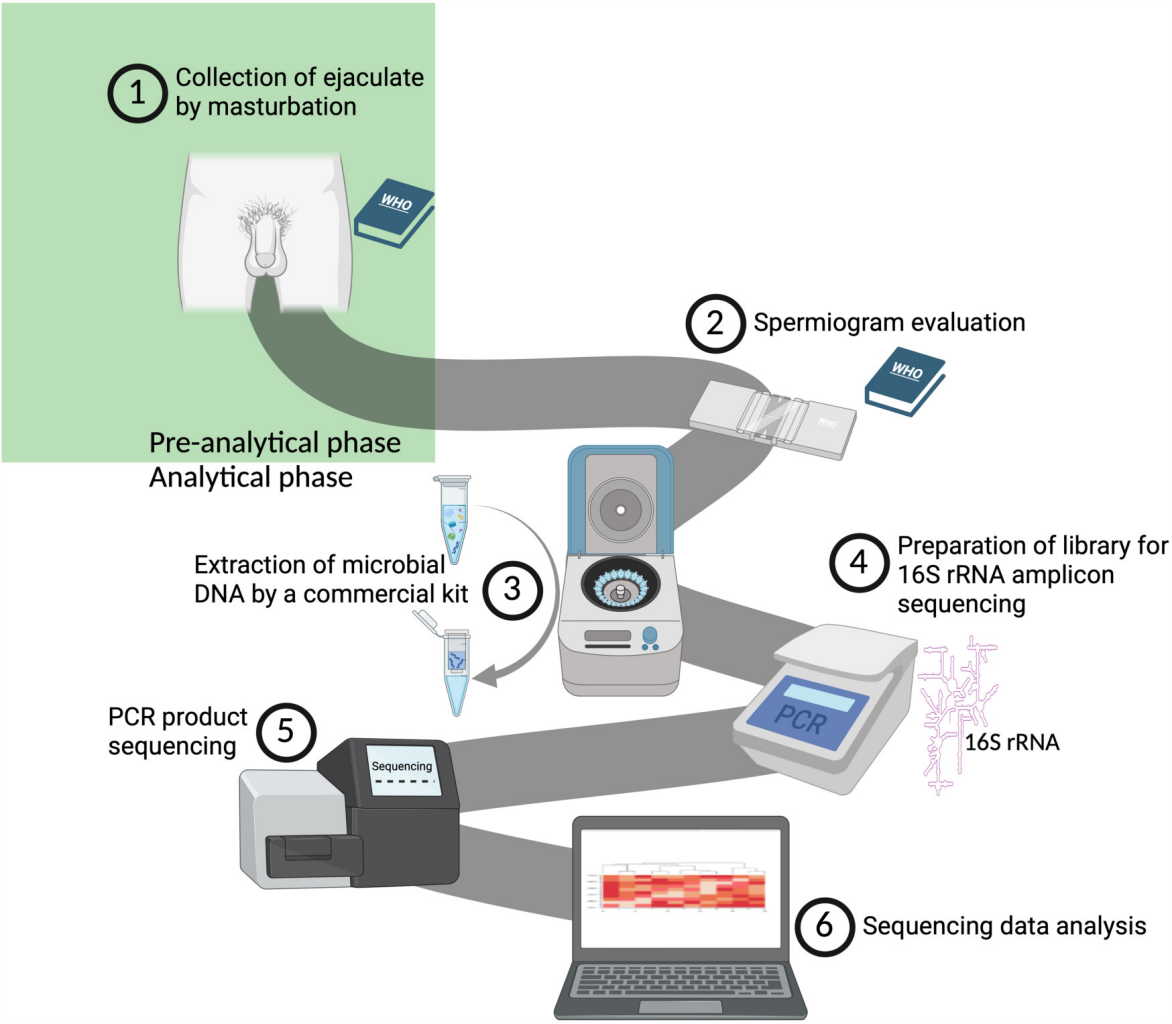
An illustration of our proposed workflow for analyzing the human seminal bacteriome (created with bioRender.com). (1) Collection of ejaculate following the guidelines outlined in the WHO Laboratory Manual for the Examination and Processing of Human Semen (61). (2) Assessment of ejaculate quality in adherence to the WHO Laboratory Manual (61). (3) Extraction of bacterial DNA using the QIAamp DNA Mini Kit (Qiagen). (4) Amplification of the gene for 16S rRNA, focusing on the V3-V4 variable region (27, 28, 35, 37, 38, 40, 60). (5) Sequencing of the amplified products on the MiSeq platform (Illumina, USA). (6) Bioinformatic and statistical analysis and interpretation of the raw data obtained.

Standardization of protocols is a central theme highlighted throughout this work. Based on the considerations discussed, it is recommended that sample collection follows WHO guidelines, particularly in maintaining a consistent abstinence period that falls within the range of 2–7 days as recommended by WHO; within a single study, however, an even narrower window should be aimed for to minimize interindividual variability. It could be assumed that the duration of abstinence towards the upper limit of the WHO range may be beneficial as it might increase the absolute amount of bacteria in the ejaculate samples. Figure 3 shows the workflow of the most commonly utilized methodical approach for analyzing the human seminal bacteriome.

Although third-generation sequencing platforms offer superior resolution and greater potential compared to second-generation technologies, their limited accessibility and adoption currently hinder their widespread use in standard workflows. The third-generation long-read sequencing should be, therefore, adopted in future studies as the technology matures and becomes more broadly available. For now, however, the authors of this review propose a standardized protocol based on short-read sequencing, which is more commonly used and supported by established workflows:

Samples should be processed within three hours of collection or stored at −80 °C to ensure sample integrity. Unless metagenomic analysis specifically requires otherwise, avoiding the separation of spermatozoa may be beneficial for microbiome analysis (especially when 16S rRNA is concerned, for which human DNA poses no interference) as partial removal of microbiota may occur during such separation. For sample pretreatment, a combined approach using bead-beating and enzymatic lysis is recommended, ideally utilizing a cocktail of lytic enzymes. DNA should then be extracted using the QIAamp DNA Mini Kit (Qiagen), which offers reliable performance in similar low-biomass samples.

For short-read sequencing, primers targeting the V3–V4 hypervariable regions of the gene for 16S rRNA appear to be the most suitable for seminal samples. Following library preparation, sequencing should be performed using the MiSeq platform (Illumina), as supported by current evidence.

To ensure data quality and monitor contamination, both positive and negative controls should be incorporated into the workflow, alongside the inclusion of a MOCK microbial community. When combined with strict adherence to good laboratory practices and, where possible, the application of appropriate bioinformatics tools, this approach should enable adequate contamination control and reproducibility in seminal microbiome studies.

## 6 Conclusion

The analysis of the human seminal bacteriome is an emerging field that has significant implications for understanding male fertility. Our review underscores the importance of methodological rigor in studying the seminal bacteriome, particularly through the amplification of the gene for 16S rRNA followed by sequencing, which has emerged as the standard approach.

However, a notable heterogeneity in the metagenomic approaches employed across studies calls for the standardization of protocols. This can facilitate performing meta-analyses combining individual studies, ultimately advancing our understanding of the role of the human seminal microbiota in fertility disorders and male reproductive health. To be able to create such standardized workflows, methodological studies comparing the performance of various protocols need to be performed. Such efforts will not only improve our understanding of the seminal microbiota but also pave the way for potential therapeutic interventions addressing male infertility.

As a standardized protocol for metagenomic analysis of human seminal bacteriome, we recommend that sample collection be performed in accordance with the WHO laboratory manual, followed by pretreatment combining mechanical disruption and enzymatic lysis. For DNA extraction, the QIAamp DNA Mini Kit (Qiagen) is advised. Library preparation should target the V3–V4 hypervariable regions of the gene for 16S rRNA, which, in our assessment, are best analyzed using the MiSeq sequencing platform (Illumina).
