# Supplementary material for: State-of-the-art approaches in the investigation of human seminal bacteriome using metagenomic methods
Source: Front Reprod Health. 2025 Jun 5;7:1557912. doi: 10.3389/frph.2025.1557912 (PMC12176750; doi:10.3389/frph.2025.1557912)
Supplement: Supplementary file 1 [file Table1.docx]

**Supplementary Table 1.** Methodological approaches utilized in studies included in our review.

| Reference | Analytical approach | Sampling method | Separation of seminal plasma and/or depletion of host DNA | Bead-beating | DNA extraction method | Region analyzed | Sequencing technology |
| --- | --- | --- | --- | --- | --- | --- | --- |
| Chen et al. (1) | 16S rRNA sequencing | Ejaculation, TESE, PESA | not performed | Y | TIANamp Genomic DNA Kit (Tiagen) | V4 | HiSeq 2000 (Illumina) |
| Monteiro et al. (2) | 16S rRNA sequencing | Ejaculation | Centrifugation | N | QIAamp DNA Mini Kit (Qiagen) | V3-V6 | Ion PGM (Thermo Fisher) |
| Baud et al. (3) | 16S rRNA sequencing | Ejaculation | not performed | Y | QIAamp DNA Mini Kit (Qiagen) | V1-V2 | MiSeq (Illumina) |
| Ricci et al. (4) | Real-Time PCR | Ejaculation | not performed | N | MagNA Pure LC DNA Kit III (Roche) | Not applied | Not applicable |
| Damke et al. (5) | Multiplex and Single target real-time PCR | Ejaculation | not performed | N | Purelink® Viral RNA/DNA Mini Kit (Thermo Fisher) | Not applied | Not applicable |
| Alfano et al. (6) | 16S rRNA sequencing | TESE | not performed | N | QiaQuick Gel Extraction Kit (Qiagen) / QIAamp DNA FFPE Tissue Kit (Qiagen) | V3-V5 | 454-GS Junior (Roche) |
| Molina et al. (7) | 16S rRNA sequencing | TESE | not performed | Y | QIAamp cador Pathogen Kit (Qiagen) | V3-V4 | MiSeq (Illumina) |
| Lundy et al. (8) | 16S rRNA sequencing | Ejaculation | QIAmp DNA Microbiome Kit (Qiagen) | Y | QIAmp PowerFecal Pro DNA Kit (Qiagen) | V3-V4 | MiSeq (Illumina) |
| Yang et al. (9) | 16S rRNA sequencing | Ejaculation | not performed | Y | CTAB method | V1-V2 | HiSeq 2500 (Illumina) |
| Okwelogu et al. (10) | 16S rRNA sequencing | Ejaculation | not performed | Y | Unspecified protocol by uBiome | V4 | MiSeq (Illumina) |
| Pagliuca et al. (11) | Multiplex real-time PCR | Ejaculation | not performed | N | RealLine DNA-Express (BIORON) | Not applied | Not applicable |
| Mørup et al. (12) | 16S rRNA sequencing | Ejaculation | Centrifugation | N | Trizol LS Reagent protocol (Invitrogen) | Not provided | HiSeq 4000 (Illumina) |
| Campisciano et al. (13) | 16S rRNA sequencing | Ejaculation | not performed | N | NucliSENS^®^ easyMAG^®^ system (BioMèrieux) | V3 | Ion PGM (Thermo Fisher) |
| Reference | **Analytical approach** | **Sampling method** | **Separation of seminal plasma and/or depletion of host DNA** | **Bead-beating** | **DNA extraction method** | **Region analyzed** | **Sequencing technology** |
| Garcia-Segura et al. (14) | 16S rRNA sequencing | Ejaculation | not performed | Y | ZymoBIOMICS DNA Microprep Kit (Zymo Research) | V1-V9 | MiSeq (Illumina) |
| Suarez Arbelaez et al. (15) | 16S rRNA sequencing | Ejaculation | not performed | N | Data not provided | V1-V2 | MiSeq (Illumina) |
| Yao et al. (16) | 16S rRNA sequencing | Ejaculation | not performed | N | QIAamp DNA Mini Kit (Qiagen) | V3-V4 | MiSeq (Illumina) |
| Bukharin et al. (17) | 16S rRNA sequencing | Ejaculation | not performed | Y | Phenol-Chloroform Extraction | Not provided | MiSeq (Illumina) |
| Chen et al. (18) | 16S rRNA sequencing | Ejaculation | not performed | N | MagPure Soil DNA Kit (TransGen Biotech) | V3-V4 | NovaSeq 6000 (Illumina) |
| Amato et al. (19) | 16S rRNA sequencing | Ejaculation | Centrifugation (for the study of ejaculate used in IVF procedure) | N | QIAamp BiOstic Bacteremia DNA Kit (Qiagen) | V3-V4 | MiSeq (Illumina) |
| Koort et al. (20) | 16S rRNA sequencing | Ejaculation | not performed | N | QIAamp DNA Blood Mini Kit (Qiagen) | V6 | HiSeq 2000 (Illumina) |
| Garcia-Segura et al. (21) | 16S rRNA sequencing | Ejaculation | not performed | Y | ZymoBIOMICS DNA Microprep Kit (Zymo Research) | V1-V9 | MinION (Oxford Nanopore Technology) |
| Štšepetova et al. (22) | 16S rRNA sequencing | Ejaculation | Centrifugation (for the study of ejaculate used in IVF procedure) | N | QIAamp DNA Mini Kit (Qiagen) / QIAamp DNA Blood Mini Kit (Qiagen) | V2-V3 | 454 FLX (Roche) |
| Yao et al. (23) | 16S rRNA sequencing | Ejaculation | not performed | N | MoBio Powersoil Isolation Kit (Qiagen) | V3-V4 | MiSeq (Illumina) |
| Osadchiy et al. (24) | 16S rRNA sequencing | Ejaculation | not performed | Y | Zymo MagBead 96 DNA/RNA kit (Zymo Research) | V1-V2 | MiSeq (Illumina) |
| Puerta Suárez et al. (25) | 16S rRNA sequencing | Ejaculation | not performed | N | Stool DNA Isolation Kit (Norgen) | V3-V4 | MiSeq (Illumina) |
| Gachet et al. (26) | 16S rRNA sequencing | Ejaculation | not performed | Y | QIAmp PowerFecal Pro DNA Kit (Qiagen) | V1-V3 | MiSeq (Illumina) |
| Reference | **Analytical approach** | **Sampling method** | **Separation of seminal plasma and/or depletion of host DNA** | **Bead-beating** | **DNA extraction method** | **Region analyzed** | **Sequencing technology** |
| Pilatz et al. (27) | Multiplex real-time PCR | Ejaculation, TESE, MESA | not performed | N | Maxwell 16 Tissue DNA Purification Kit (Promega) | Not applied | Not applicable |
| Rivera et al. (28) | Multiplex real-time PCR | Ejaculation | not performed | N | Phenol-Chloroform Extraction | Not applied | Not applicable |
| Veneruso et al. (29) | 16S rRNA sequencing | Ejaculation | not performed | N | Phenol-Chloroform Extraction | V4-V6 | MiSeq (Illumina) |

TESE,Testicular Sperm Extraction; PESA, Percutaneous Sperm Aspiration; MESA, Microepididymal Sperm Aspiration; CTAB, Cetrimonium Bromide-based extraction; Y, yes; N, no

**References**

1. Chen H, Luo T, Chen T, Wang G. Seminal bacterial composition in patients with obstructive and non‑obstructive azoospermia. Exp Ther Med [Internet]. 2018 Jan 19 [cited 2024 Oct 10]; Available from: http://www.spandidos-publications.com/10.3892/etm.2018.5778

2. Monteiro C, Marques PI, Cavadas B, Damião I, Almeida V, Barros N, et al. Characterization of microbiota in male infertility cases uncovers differences in seminal hyperviscosity and oligoasthenoteratozoospermia possibly correlated with increased prevalence of infectious bacteria. Am J Reprod Immunol. 2018 Jun;79(6):e12838.

3. Baud D, Pattaroni C, Vulliemoz N, Castella V, Marsland BJ, Stojanov M. Sperm Microbiota and Its Impact on Semen Parameters. Front Microbiol. 2019 Feb 12;10:234.

4. Ricci S, De Giorgi S, Lazzeri E, Luddi A, Rossi S, Piomboni P, et al. Impact of asymptomatic genital tract infections on in vitro Fertilization (IVF) outcome. Manganelli R, editor. PLOS ONE. 2018 Nov 16;13(11):e0207684.

5. Damke E, Kurscheidt FA, Irie MMT, Gimenes F, Consolaro MEL. Male Partners of Infertile Couples With Seminal Positivity for Markers of Bacterial Vaginosis Have Impaired Fertility. Am J Mens Health. 2018 Nov;12(6):2104–15.

6. Alfano M, Ferrarese R, Locatelli I, Ventimiglia E, Ippolito S, Gallina P, et al. Testicular microbiome in azoospermic men—first evidence of the impact of an altered microenvironment. Hum Reprod. 2018 Jul 1;33(7):1212–7.

7. Molina NM, Plaza-Díaz J, Vilchez-Vargas R, Sola-Leyva A, Vargas E, Mendoza-Tesarik R, et al. Assessing the testicular sperm microbiome: a low-biomass site with abundant contamination. Reprod Biomed Online. 2021 Sep;43(3):523–31.

8. Lundy SD, Sangwan N, Parekh NV, Selvam MKP, Gupta S, McCaffrey P, et al. Functional and Taxonomic Dysbiosis of the Gut, Urine, and Semen Microbiomes in Male Infertility. Eur Urol. 2021 Jun;79(6):826–36.

9. Yang H, Zhang J, Xue Z, Zhao C, Lei L, Wen Y, et al. Potential Pathogenic Bacteria in Seminal Microbiota of Patients with Different Types of Dysspermatism. Sci Rep. 2020 Apr 23;10(1):6876.

10. Okwelogu SI, Ikechebelu JI, Agbakoba NR, Anukam KC. Microbiome Compositions From Infertile Couples Seeking In Vitro Fertilization, Using 16S rRNA Gene Sequencing Methods: Any Correlation to Clinical Outcomes? Front Cell Infect Microbiol. 2021 Oct 1;11:709372.

11. Pagliuca C, Cariati F, Bagnulo F, Scaglione E, Carotenuto C, Farina F, et al. Microbiological Evaluation and Sperm DNA Fragmentation in Semen Samples of Patients Undergoing Fertility Investigation. Genes. 2021 Apr 27;12(5):654.

12. Mørup N, Main AM, Jørgensen N, Daugaard G, Juul A, Almstrup K. The seminal plasma microbiome of men with testicular germ cell tumours described by small RNA sequencing. Andrology. 2023 May;11(4):756–69.

13. Campisciano G, Iebba V, Zito G, Luppi S, Martinelli M, Fischer L, et al. Lactobacillus iners and gasseri, Prevotella bivia and HPV Belong to the Microbiological Signature Negatively Affecting Human Reproduction. Microorganisms. 2020 Dec 25;9(1):39.

14. Garcia-Segura S, Del Rey J, Closa L, Garcia-Martínez I, Hobeich C, Castel AB, et al. Seminal Microbiota of Idiopathic Infertile Patients and Its Relationship With Sperm DNA Integrity. Front Cell Dev Biol. 2022 Jun 28;10:937157.

15. Suarez Arbelaez MC, Israeli JM, Tipton CD, Loloi J, Deebel N, Leong JY, et al. Pilot Study: Next-generation Sequencing of the Semen Microbiome in Vasectomized Versus Nonvasectomized Men. Eur Urol Focus. 2023 Jan;9(1):75–82.

16. Yao Y, Qiu XJ, Wang DS, Luo JK, Tang T, Li YH, et al. Semen microbiota in normal and leukocytospermic males. Asian J Androl. 2022 Jul;24(4):398–405.

17. Bukharin OV, Perunova NB, Ivanova EV, Chaynikova IN, Bekpergenova AV, Bondarenko TA, et al. Semen microbiota and cytokines of healthy and infertile men. Asian J Androl. 2022 Jul;24(4):353–8.

18. Chen P, Li Y, Zhu X, Ma M, Chen H, He J, et al. Interaction between Host and Microbes in the Semen of Patients with Idiopathic Nonobstructive Azoospermia. Hung YP, editor. Microbiol Spectr. 2023 Feb 14;11(1):e04365-22.

19. Amato V, Papaleo E, Pasciuta R, Viganò P, Ferrarese R, Clementi N, et al. Differential Composition of Vaginal Microbiome, but Not of Seminal Microbiome, Is Associated With Successful Intrauterine Insemination in Couples With Idiopathic Infertility: A Prospective Observational Study. Open Forum Infect Dis. 2020 Jan 1;7(1):ofz525.

20. Koort K, Sõsa K, Türk S, Lapp E, Talving E, Karits P, et al. *Lactobacillus crispatus* ‐dominated vaginal microbiome and *Acinetobacter* ‐dominated seminal microbiome support beneficial ART outcome. Acta Obstet Gynecol Scand. 2023 Jul;102(7):921–34.

21. Garcia-Segura S, Del Rey J, Closa L, Garcia-Martínez I, Hobeich C, Castel AB, et al. Characterization of Seminal Microbiome of Infertile Idiopathic Patients Using Third-Generation Sequencing Platform. Int J Mol Sci. 2023 Apr 26;24(9):7867.

22. Štšepetova J, Baranova J, Simm J, Parm Ü, Rööp T, Sokmann S, et al. The complex microbiome from native semen to embryo culture environment in human in vitro fertilization procedure. Reprod Biol Endocrinol. 2020 Dec;18(1):3.

23. Yao T, Han X, Guan T, Wang Z, Zhang S, Liu C, et al. Effect of indoor environmental exposure on seminal microbiota and its application in body fluid identification. Forensic Sci Int. 2020 Sep;314:110417.

24. Osadchiy V, Belarmino A, Kianian R, Sigalos JT, Ancira JS, Kanie T, et al. Semen microbiota are dramatically altered in men with abnormal sperm parameters. Sci Rep. 2024 Jan 11;14(1):1068.

25. Puerta Suárez J, Cardona Maya WD. Microbiota, Prostatitis, and Fertility: Bacterial Diversity as a Possible Health Ally. Ather MH, editor. Adv Urol. 2021 Sep 28;2021:1–8.

26. Gachet C, Prat M, Burucoa C, Grivard P, Pichon M. Spermatic Microbiome Characteristics in Infertile Patients: Impact on Sperm Count, Mobility, and Morphology. J Clin Med. 2022 Mar 9;11(6):1505.

27. Pilatz A, Kilb J, Kaplan H, Fietz D, Hossain H, Schüttler CG, et al. High prevalence of urogenital infection/inflammation in patients with azoospermia does not impede surgical sperm retrieval. Andrologia [Internet]. 2019 Nov [cited 2024 Nov 13];51(10). Available from: https://onlinelibrary.wiley.com/doi/10.1111/and.13401

28. Rivera VV, Cardona Maya WD, Puerta-Suárez J. The relationship between sexually transmitted microorganisms and seminal quality in asymptomatic men. Asian J Urol. 2022 Oct;9(4):473–9.

29. Veneruso I, Cariati F, Alviggi C, Pastore L, Tomaiuolo R, D’Argenio V. Metagenomics Reveals Specific Microbial Features in Males with Semen Alterations. Genes. 2023 Jun 6;14(6):1228.
